# Continuous Picking Path Planning Based on Lightweight Marigold Corollas Recognition in the Field

**DOI:** 10.3390/biomimetics10100648

**Published:** 2025-09-26

**Authors:** Baojian Ma, Zhenghao Wu, Yun Ge, Bangbang Chen, Jijing Lin, He Zhang, Hao Xia

**Affiliations:** 1 Department of Mechanical and Electrical Engineering, Xinjiang Institute of Technology, Aksu 843100, China; 11813019@zju.edu.cn (B.M.); chenbangbang@st.xatu.edu.cn (B.C.); 2023274@xjit.edu.cn (J.L.); 2 College of Mechanical and Electrical Engineering, Shihezi University, Shihezi 832003, China; gy_mac@shzu.edu.cn (Y.G.); zhangh@stu.shzu.edu.cn (H.Z.); xiahao@stu.shzu.edu.cn (H.X.)

**Keywords:** marigold, identification, lightweight, continuous picking, path planning, automated harvesting

## Abstract

This study addresses the core challenges of precise marigold corollas recognition and efficient continuous path planning under complex natural conditions (strong illumination, occlusion, adhesion) by proposing an integrated lightweight visual recognition and real-time path planning framework. We introduce MPD-YOLO, an optimized model based on YOLOv11n, incorporating (1) a Multi-scale Information Enhancement Module (MSEE) to boost feature extraction; (2) structured pruning for significant model compression (final size: 2.1 MB, 39.6% of original); and (3) knowledge distillation to recover accuracy loss post-pruning. The resulting model achieves high precision (P: 89.8%, mAP@0.5: 95.1%) with reduced computational load (3.2 GFLOPs) while demonstrating enhanced robustness in challenging scenarios—recall significantly increased by 6.8% versus YOLOv11n. Leveraging these recognition outputs, an adaptive ant colony algorithm featuring dynamic parameter adjustment and an improved pheromone strategy reduces average path planning time to 2.2 s—a 68.6% speedup over benchmark methods. This integrated approach significantly enhances perception accuracy and operational efficiency for automated marigold harvesting in unstructured environments, providing robust technical support for continuous automated operations.

## 1. Introduction

Marigold (*Tagetes erecta* L.) is an annual herb of the compositae family. Its flowers are rich in volatile oils, flavonoids, polysaccharides, triterpenoids and various other essential trace elements for the human body, and have significant value in horticultural appreciation, healthcare and food processing [1]. However, the current manual harvesting method (low efficiency, high cost and significant damage) has become a key bottleneck restricting its industrial development. The harvesting window period of this crop is concentrated from July to October, and harvesting operations need to be carried out approximately every 10 days. The existing harvesting mode has high labor intensity and low efficiency, resulting in high costs and inability to meet the needs of large-scale planting. Therefore, the development of efficient, precise and low-damage harvesting technology is crucial for promoting the sustainable development of the marigold industry. Existing mechanical harvesters typically employ non-selective strategies, which generally have problems such as low cleaning rate and severe mechanical damage to the plants [2]. With the rapid development of artificial intelligence and robot technology, agricultural robots provide a new technical path for the realization of automatic and precise harvesting of marigolds. The core challenge lies in the high-precision recognition of target flowers and, based on this recognition, efficient continuous path planning. Therefore, achieving accurate marigold target recognition is the primary and crucial step.

However, the realization of this crucial step faces significant challenge. Traditional image processing methods are significantly constrained in their application scope due to their reliance on manual feature design, high environmental sensitivity, lack of semantic understanding capabilities, and difficulty in adapting to complex scenarios [3,4]. In recent years, the rise in deep learning technology has driven research on flower recognition based on point clouds and image segmentation. However, the inherent challenges of 3D point cloud technology, such as large data volume and high engineering costs, limit its practical application for apical flower recognition [5]. Consequently, lightweight 2D image-based deep learning models have emerged as a more practical research direction. Zhang et al. [6] detected and localized red flower filaments using an enhanced DeepLabv3+ with ShuffleNetV2 backbone and multi-rate convolutions for feature extraction. The model achieved 92.50% average localization and 90.83% picking success, compressing size from 206.36 MB to 25.54 MB for effective lightweighting. Similarly, Zhang et al. [7] employed the YOLOv8s-seg network for locating picking points during the full bloom of red flowers, achieving a segmentation accuracy, recall rate, and mean mAP of 89.1%, 79.9%, and 85.7%, respectively. However, the model’s size (34.9 MB) and computational complexity (49.5 GFLOPs) remain substantial. Overall, although existing top flower picking point recognition methods based on segmentation models have achieved results, the problem of excessive model size and parameter count remains largely unsolved, which restricts their efficient deployment on edge computing platforms.

The YOLO series models have increasingly attracted academic attention due to their high efficiency and lightweight nature in flower recognition tasks. For instance, Qi et al. [8] proposed MC-LCNN, a lightweight CNN for medicinal chrysanthemum detection. It combines an MC-ResNetv1 backbone with an MC-ResNetv2 neck using FPN fusion, demonstrating high robustness in complex environments and achieving 93.06% accuracy. Similarly, Bai et al. [9] introduced a GS-ELAN neck into YOLOv7, suppressing noise from high-resolution heads. Integrating a Swin Transformer head further improved performance, achieving 93.9% accuracy, 93% recall, and 94.7% mAP for strawberry flower detection. These studies collectively validate the effectiveness of strategic module replacement and network enhancement in improving target recognition performance. Customized network improvements for specific applications (such as chrysanthemum detection) have also shown significant results: Hee-Mun Park et al. [10] proposed a lightweight YOLOv4-based chrysanthemum detection model. Employing a CSPDarknet53-Tiny backbone with FPN and a context bounding box mechanism, the model achieved a 96.62% average recognition rate. Zhao et al. [11] enhanced the CR-YOLOv5s model for chrysanthemum flower cluster detection by replacing the backbone convolutional module with RepVGG and integrating Coordinate Attention into the feature pyramid, boosting mAP to 93.9%, significantly higher than the baseline. However, while these studies improve accuracy through module augmentation or attention mechanisms, many incur substantial computational costs, limiting deployment on resource-constrained edge devices.

To meet the demands of lightweight and efficient deployment of models in complex field environments, researchers have actively explored various technical paths, among which improving the network structure is a common strategy. In the detection of flowering periods in complex environments, Qi et al. [12] proposed TC-YOLO for chrysanthemum detection, which achieved 92.49% accuracy with real-time inference speed of 47.23 FPS, demonstrating strong edge deployment potential. Shang et al. [13] suggested an enhanced YOLOv5s model for apple blossom thinning, achieving 88.40% accuracy and 91.80% AP with a compact model size of 0.61 MB and 86.21 FPS inference speed. Although lightweight models are validated for efficacy, such compression often compromises accuracy. In the area of network optimization, Chen et al. [14] presented the lightweight YOLO-SaFi algorithm, which reduced the model size, computational complexity, and parameter count by 48.2%, 40.7%, and 50.0%, respectively, compared to the original model, while significantly improving detection speed. To address the accuracy compromise associated with network optimization, Zhang et al. [15] developed WED-YOLO, a YOLOv8-based model for safflower recognition. By introducing a dynamic upsampling layer, adding a small object detection layer, and optimizing the loss function, they achieved 93.15% recognition accuracy and 89.64% average precision. Beyond direct network architecture optimization, model compression techniques—such as pruning and knowledge distillation—are equally critical for achieving model lightweightness. Knowledge distillation can effectively enhance accuracy while controlling model size: Fatehi et al. [16] employed offline knowledge distillation to optimize the YOLOv9t model, transferring key features from teacher to student and increasing Damascus rose recognition accuracy to 96.2% with a model size of only 4.43 MB. Lyu et al. [17] combined multi-teacher pre-activation features and a LogCoshSquared loss for distillation, which enabled rapid lychee flower identification. The compressed model (5.91 MB) achieved 94.21% average precision on an embedded platform. Additionally, Fan et al. [18] developed a high-precision, lightweight model (15.04 MB) for marigold corolla detection by replacing standard convolution with depthwise separable convolution (DSConv), simplifying the SPP module in YOLOv7, and combining pruning with fine-tuning. Despite significant advancements in model compression techniques—including architectural improvements, knowledge distillation, and pruning—to reduce model size and enhance efficiency, the current lightweight models still exhibit insufficient recognition robustness when confronted with complex field scenarios such as dense marigold cultivation, severe occlusion, and overlapping corollas. Moreover, the size of existing models often necessitates further compression to meet the stringent resource constraints of low-cost field robotic platforms operating on edge devices. These limitations in recognition robustness and model size critically impede the implementation of robust recognition systems, which are a prerequisite for efficient continuous path planning.

Furthermore, the development of such planning strategies is itself a critical challenge, particularly for crops like marigolds characterized by dense growth, occlusion, and adhesion. Unfortunately, dedicated research on continuous picking path planning for marigolds remains scarce. Existing studies have mainly focused on vertically grown plants such as safflower and tea bushes. Zhang et al. [19] proposed an improved ant colony algorithm (ACO) for 3-D safflower picking-path planning. By optimizing secondary paths, their method reduced the number of visited points by three, shortened the total path length by 74.32%, and cut picking time by 0.957 s compared with the benchmark. In tea harvesting, Wu et al. [20] introduced an improved ant colony algorithm (IACA) featuring an adaptive pheromone evaporation mechanism to mitigate susceptibility to local optima. Relative to the benchmark and other comparable algorithms, IACA reduced the planned path length by 6% and cut the number of iterations required for convergence by 60%. Wang et al. [21] further incorporated a regional division strategy: the tea garden was first partitioned into zones, after which an improved ACO—with adaptive termination and path reuse for initialization—was applied to plan paths within each zone, significantly decreasing zone-wise planning time. In contrast, the dense, horizontally spreading habit of marigolds often leads to mutual occlusion and flower adhesion, posing distinct challenges for continuous and efficient picking-path planning. Consequently, adapting existing ant colony-based strategies to the occlusion-prone morphology of marigolds—especially when the recognition model itself must be extremely lightweight—remains an unresolved challenge.

More critically, these two aspects—accurate yet lightweight flower recognition and occlusion-aware path planning—are rarely studied in a coupled manner; the former directly determines the spatial priors available to the latter, while the latter’s efficiency feedback constrains the permissible complexity of the former. Therefore, this study addresses critical challenges in automated marigold harvesting within natural environments by proposing a novel lightweight recognition model. Building upon this, we develop a continuous path planning strategy for marigold picking. The main contributions of this paper are as follows:

(1) To enhance target recognition in the YOLOv11n model, we propose enhancements to the core C3k2 module, integrating an edge enhancement mechanism and a multi-scale fusion strategy. This improves extraction and fusion of multi-scale edge features in natural scenes.
(2) To optimize model deployment efficiency, this study proposes a compression strategy integrating structured pruning and knowledge distillation. This approach significantly reduces model parameters and complexity while maintaining recognition accuracy, achieving efficient compression performance co-optimization.
(3) Leveraging the real-time flower recognition generated by our compressed YOLOv11n model as dynamic occupancy priors, we further propose an improved ant colony algorithm. This approach reduces the time to find optimal paths and significantly enhances harvesting efficiency.

## 2. Materials and Methods

### 2.1. Collection and Preparation of Marigold Data

**Figure 1:**
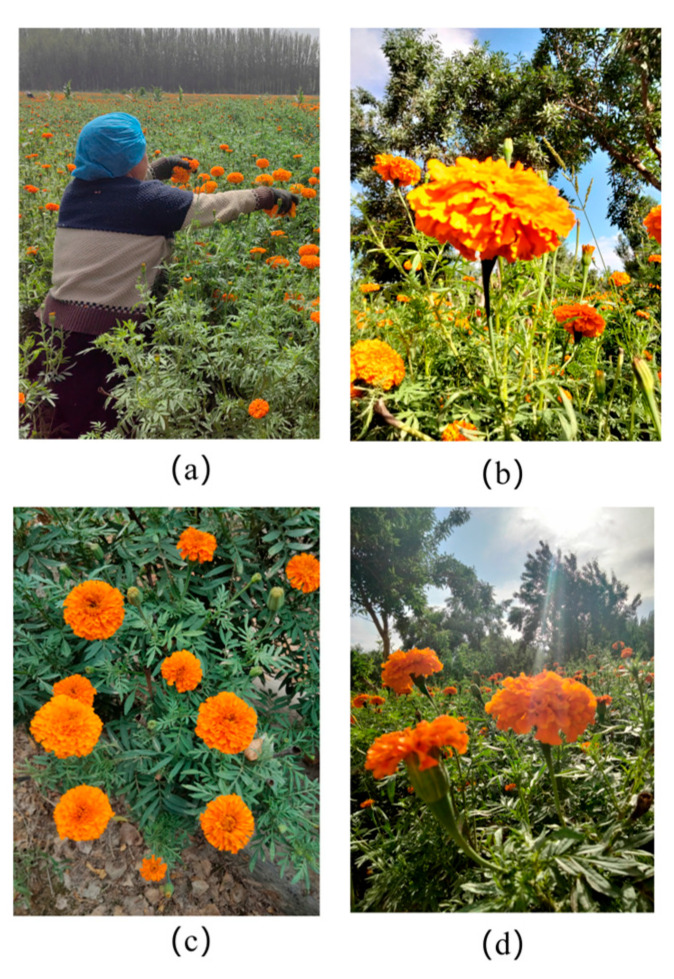
Sample data of marigold: (**a**) large-scale scene; (**b**) side view; (**c**) front lighting; (**d**) back lighting.

The study area was a marigold planting base located in Yingwustang Township, Shache County, Kashgar Prefecture, Xinjiang, where a ridge planting pattern was adopted to facilitate subsequent mechanical harvesting (Figure 1a). Under natural light conditions, mature marigold plants in full bloom were imaged using a Huawei P20 camera (Huawei Technologies Co., Ltd., Shenzhen, China) from multiple angles, covering side view, front lighting, and back lighting scenarios (Figure 1b–d). A total of 3000 digital images (3120 × 4160 pixels) were collected. The dataset was partitioned into training (2100 images), validation (600 images), and test (300 images) sets at a 7:2:1 ratio. Using the LabelImg annotation tool, visible and non-severely occluded marigold corollas were annotated in all images with rectangular bounding boxes; severely occluded individuals were excluded. This process generated annotation files in the required format for training and testing the YOLO object detection model.

### 2.2. Method Overview

**Figure 2:**
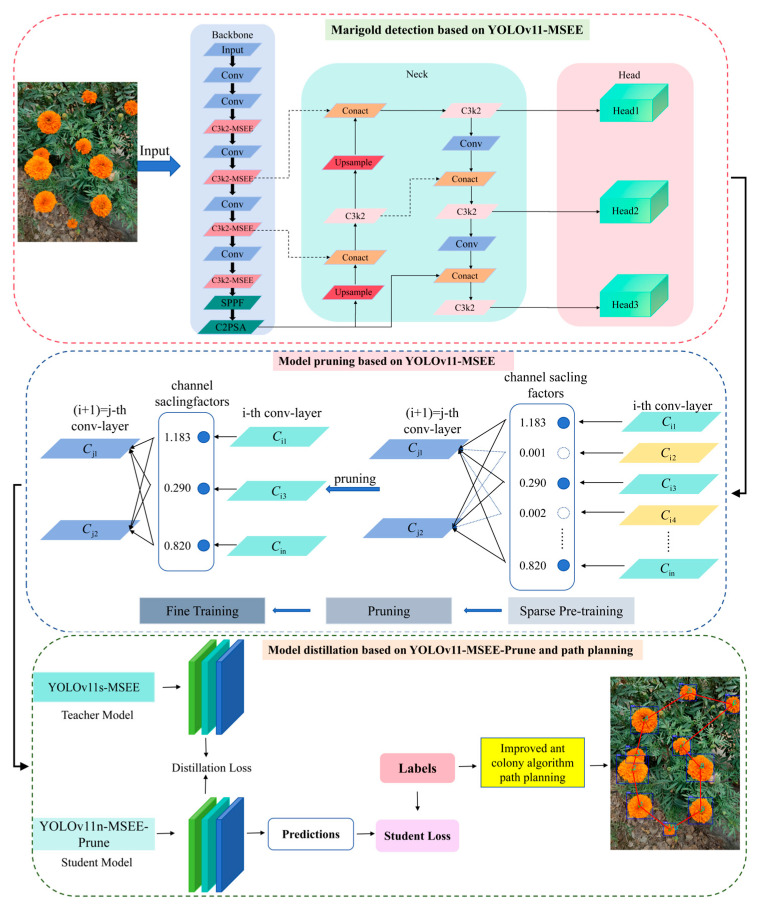
The flow of the proposed method.

Marigold images covering diverse lighting conditions (front, back, side light) and complex scenarios (dense crown adhesion, leaf occlusion) were collected from large-scale plantations and batch-annotated to construct the training dataset. Based on the YOLO v11n architecture, we incorporated the Multi-Scale Edge Information Enhancement module (MSEE) [22] to enhance the model’s ability to recognize occluded targets within complex backgrounds, and further optimized the model via pruning and knowledge distillation; the overall framework of the proposed lightweight marigold recognition model (MPD-YOLO) and continuous harvesting path planning approach is shown in Figure 2. Furthermore, to achieve lightweight deployment on edge devices, channel pruning was applied to the fully trained model. This effectively removed redundant channels while maximally preserving key feature extraction capabilities, significantly reducing model size and parameter count. To mitigate potential accuracy loss from pruning, a knowledge distillation strategy is employed. Here, a high-performance teacher model guides the pruned student model, effectively transferring its superior feature representation capabilities. This enhances detection accuracy without increasing model complexity. Finally, to improve continuous harvesting efficiency, the picking path is optimized algorithmically for continuous path planning.

### 2.3. Improvements of the YOLOv11n Mode

Accurate extraction of multi-scale edge features presents a significant challenge for improving the object recognition performance. While the C3k2 module in the YOLOv11 architecture effectively reduces computational complexity and parameter count through efficient feature extraction and fusion, its reliance on traditional single-scale feature extraction limits its ability to concurrently model fine-grained edges of small objects and the global contours of large objects. Furthermore, the inherent smoothing characteristic of convolution operations can lead to the blurring of critical edge details. To address the precise recognition and localization of chrysanthemums in complex environments, this study proposes an enhanced C3k2 module and integrates a novel Multi-Scale Edge Information Enhancement (MSEE) module, as illustrated in the first part of Figure 2. This integrated approach aims to strengthen the model’s capacity for multi-scale perception and representation of salient features.

#### 2.3.1. Lightweight Module C3k2-MSEE

**Figure 3:**
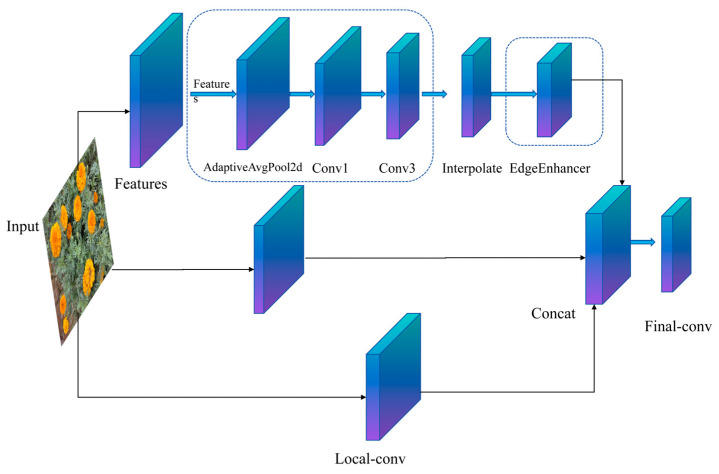
The lightweight module C3k2-MSEE.

Figure 3 illustrates the architecture of the proposed Multi-scale Edge Information Enhancement (MSEE) module, which synergistically integrates multi-scale perception with edge refinement for efficient edge feature modeling. The module comprises three key components: (1) a multi-scale feature perception branch employing adaptive average pooling to generate multi-resolution feature maps, followed by 1 × 1 convolution for channel reduction and 3 × 3 depthwise separable convolution for efficient cross-scale feature extraction; (2) an edge enhancement sub-module that computes spatial gradients (e.g., via Sobel operators) on pooled features to highlight edge transitions, refines them convolutionally, and integrates the enhanced edges with the original input via residual summation, significantly improving the edge signal-to-noise ratio while suppressing noise; and (3) a local-to-global fusion mechanism where a dedicated 3 × 3 convolution branch preserves high-resolution texture details, multi-scale features are upsampled and concatenated channel-wise with these local features, and a final convolutional layer integrates them into a unified representation, achieving complementary fusion of fine-grained details and contextual information.

#### 2.3.2. Model Pruning

**Figure 4:**
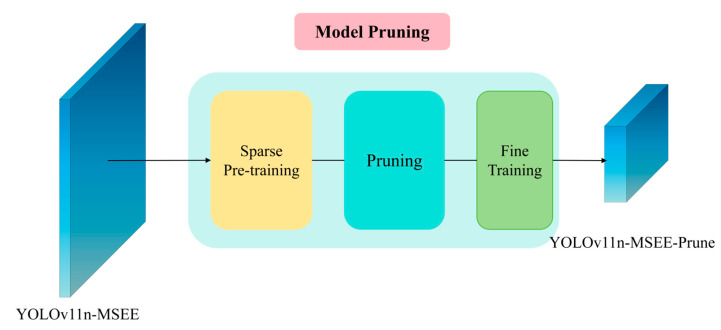
YOLOv11n-MSEE-Prune.

Figure 4 illustrates the model-pruning algorithm adopted in this study. Model pruning is a deep learning compression technique whose core idea is to remove redundant or unimportant parameters—such as weights, channels, neurons, or layers—from the neural network. While preserving a baseline level of accuracy on marigold recognition, the technique reduces the model’s parameter count, computational complexity, and storage footprint, thereby improving inference efficiency and lowering the cost of edge deployment. In this work, we employ a prune-and-fine-tune strategy [23]. The procedure is as follows. First, the model undergoes sparse training for a fixed number of epochs with L1-regularization to induce sparsity in the weight distribution, preparing the network for pruning. Second, we apply Layer-adaptive Magnitude-based Pruning (LAMP) [24] to identify and remove weights that contribute least to the model’s output. Finally, the pruned model is fine-tuned to optimize the remaining parameters and to restore, or even enhance, its original performance. The detailed LAMP algorithm is presented below:

(1) $$Score=\alpha \cdot \left(\frac{\left|W_{c}\right|}{\sqrt{\sum_{c^{\prime }\in R}{\left|W_{c^{\prime }}\right|}^{2}}}\right)+(1-\alpha ).SpeedUpPenalty(c)$$

Here, *c* represents the set of all channels in the current layer, and $W_{c}$ is the weight tensor of the *c*-th channel. The weights of the convolutional layer are extracted through Detection Compressor. During the pruning process, setting global pruning to true can trigger the Lamp score sorting of all channels in the entire model, ensuring that the pruned model retains the global key features. SpeedUpPenalty(c) is the speed improvement penalty term, used to constrain the inference speed of the pruned model. Its formula is

(2) $$SpeedUpPenalty(c)=\beta \cdot \left(\frac{Flops(c)}{Flops_{t\arg et}}-1\right)$$

Among them, Flops(c) represents the parameter quantity of the *c*-th channel after pruning; $Flops_{t\arg et}$ represents the target parameter quantity, and the target speed is set to 2.0, that is, $Flops_{t\arg et}$ is 50% of the original model’s parameter quantity; and α,β represents the balance coefficient, which is controlled by the hyperparameter of the sparse training.

#### 2.3.3. Model Distillation

**Figure 5:**
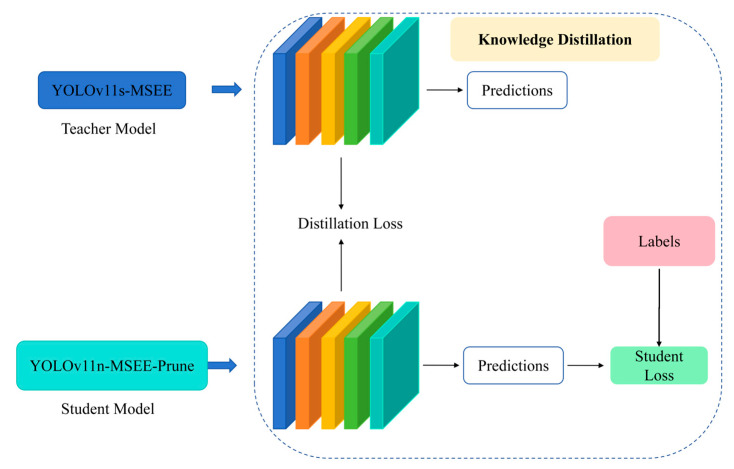
Distillation Algorithm.

As depicted in Figure 5, this study employs Knowledge Distillation (KD) for model compression and knowledge transfer. In KD, knowledge is transferred from a complex teacher model to a lightweight student model. This transfer aims to enhance the student’s performance and generalization capacity without increasing its parameter count or architectural complexity, thereby reducing computational costs and facilitating edge deployment. A multi-dimensional distillation strategy integrates feature distillation—which guides the student model to learn intermediate teacher representations (e.g., spatial structures and semantic features) using Correlation Weighted Distillation (CWD) [25] that transfers inter-feature dependencies via global correlation matrices—and logit distillation, which aligns student outputs with the teacher’s predictive distribution through soft labels to propagate inter-class relational information. The distillation loss function comprises three constituent components:

(3) $$L_{total}=L_{task}+\alpha \cdot L_{\log ical}+\beta \cdot L_{feature}$$

among them, $L_{task}$ is the original detection task loss of the student model; α is the weight of the logical layer loss; and β is the weight of the feature layer loss.

### 2.4. Path Planning

To optimize the efficiency of marigold automatic harvesting in complex environments, this study proposes continuous picking path planning based on an improved ant colony algorithm. As a general search technique inspired by swarm intelligence, the ant colony algorithm is well-suited for solving complex combinatorial optimization problems. Efficient and rapid harvesting operations necessitate rational planning of the continuous picking sequence. The proposed algorithm initiates with a randomly generated starting point and, upon convergence to the global optimum, effectively constrains picking trajectory. Specifically, *m* artificial ants are deployed within the target environment containing *n* marigold corollas. Each ant probabilistically selects the next unvisited target location based on the accumulated pheromone concentration and heuristic information along the path, ensuring no revisitation occurs. Upon visiting all targets, each ant returns to its origin, thereby completing a closed tour. The system evaluates all constructed paths and selects the optimal picking sequence for execution. The core of the algorithm lies in the transition probability $P_{ij}^{k}$ of ant *k* choosing the next target point *j* from the current position *i*, which is defined as follows:

(4) $$P_{ij}^{k}=\left\{\begin{array}{l} \frac{\tau_{ij}^{\partial }\left(t\right)\eta_{ij}^{\beta }\left(t\right)}{\sum_{s\in allowed_{k}}\tau_{is}^{\partial }\left(t\right)\eta_{is}^{\beta }\left(t\right)},j\in allowed_{k}\\ 0,otherwise \end{array}\right.$$

here, $\tau_{ij}$ represents the pheromone intensity on the edge $\left(i,j\right)$ which reflects the prior experience of the ant colony on this edge and is the amount of information accumulated by the ant colony during the optimization process; $\eta_{ij}$ is the visibility on the edge $\left(i,j\right)$, which reflects the heuristic information during the movement of ants; α is the pheromone importance factor, which reflects the intensity of the random factor’s effect in the path search by the ant colony; and β is the visibility heuristic factor, and its magnitude reflects the intensity of the deterministic factor’s effect in the path search by the ant colony.

**Figure 6:**
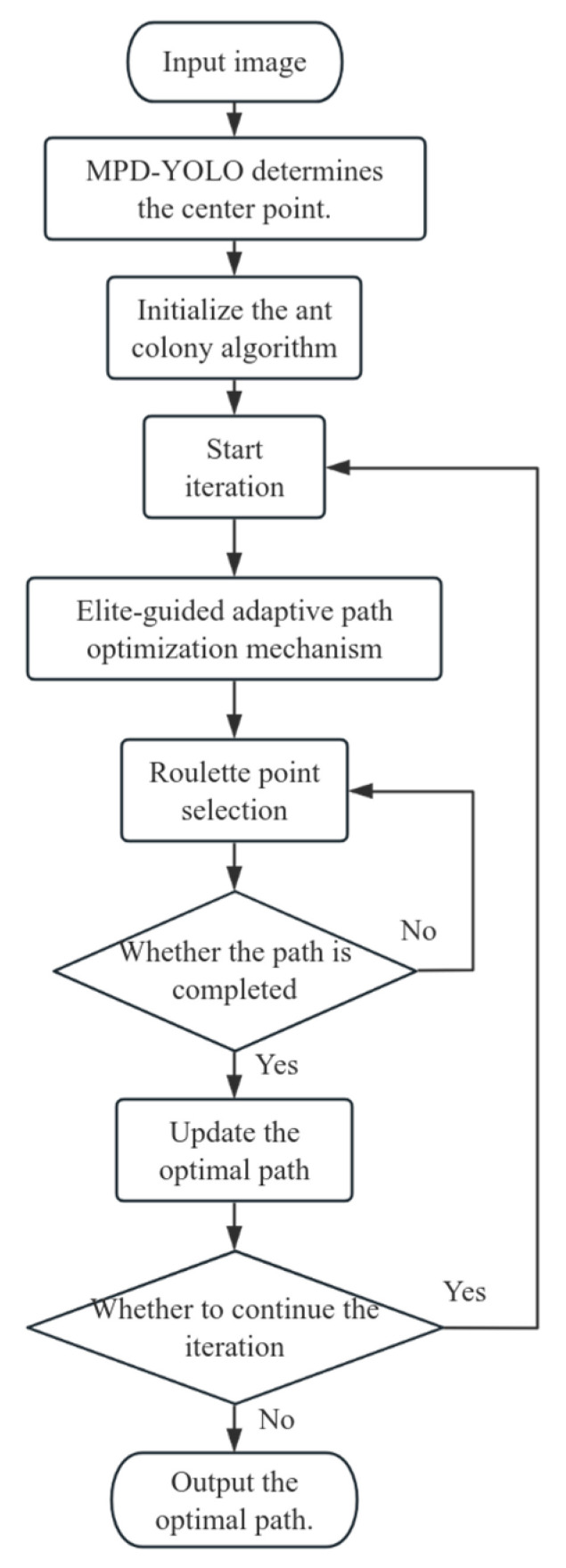
Flowchart of the Improved Ant Colony Algorithm.

The traditional ant colony optimization (ACO) algorithm exhibits limitations in convergence speed and susceptibility to premature convergence, often becoming trapped in local optima. These drawbacks are particularly acute for large-scale problems, where the substantial number of iterations required to reach the optimal solution struggles to meet the efficiency demands of complex scenarios. To mitigate these issues, this study introduces a parameter adaptive mechanism, dynamically adjusting key parameters (α,β,ρ) based on iteration progress, and an elite pheromone update strategy, which selectively reinforces only the pheromone trails of the current global-best path to suppress ineffective diffusion [26]. These enhancements collectively accelerate convergence, yield shorter planned path lengths, reduce the need for frequent visualization and multiple trial averaging, and effectively curtail computational costs while ensuring solution stability. The specific implementation process is detailed in Figure 6.

### 2.5. Experimental Environment and Parameter Settings

This study was conducted on a platform running the Microsoft Windows 10 operating system. The hardware configuration comprised an Intel (R) Core (TM) i9-14900KF central processing unit (CPU) and an NVIDIA GeForce RTX 4070 Super graphics processing unit (GPU) equipped with 16 GB of video memory. The software environment utilized Python 3.11, with the deep learning framework implemented using the PyTorch 2.0.0 library accelerated by Compute Unified Device Architecture (CUDA) 11.8. During the initial model training phase, a batch size of 32, 4 data loading threads, and 300 epochs of training were employed. Optimization was performed using the Stochastic Gradient Descent (SGD) algorithm, with the final model selected based on validation set performance. For the subsequent model pruning stage, training proceeded for 300 epochs with a reduced batch size of 16 and 4 data loading threads, applying the Layer-adaptive Magnitude Pruning (LAMP) method. In the knowledge distillation stage, the batch size was reverted to 32 and the number of data loading threads was increased to 8. This comprehensive optimization pipeline culminated in the acquisition of the target data model.

### 2.6. Model Evaluation Indicators

To evaluate the performance and parameters of the improved model, the following indicators are adopted: for the recognition accuracy of marigolds, Precision (P), Recall (R), and mean Average Precision (mAP) are used for assessment; for the recognition speed and model complexity, Parameters, Floating Point Operations (FLOPs), and Model Size are employed for measurement; and for the path planning effect, the average path length, average running time, and optimal path length are utilized for evaluation.

## 3. Results and Analysis

### 3.1. Evaluation of Training Results

**Figure 7:**
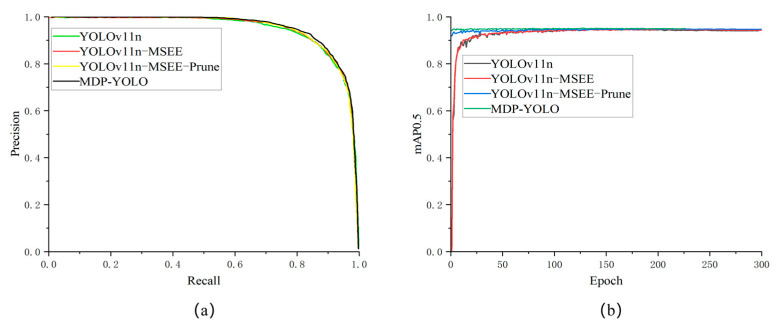
Comparison among various curves during the training process; (**a**) mAP0.5 curve; (**b**) PR curve.

Figure 7a presents the precision-recall (PR) curves and mAP@0.5 curves of the baseline model (YOLOv11n), the improved model (YOLOv11n-MSEE), the pruned model (YOLOv11n-MSEE-Prune), and the pruned-distilled model (MPD-YOLO). Firstly, by comparing the PR curves of the four models (which assess the classifier’s performance by depicting the relationship between precision and recall at different prediction thresholds, and a larger area under the curve indicates better model performance), it can be seen that although the area under each model’s curve is relatively large, the PR curve of the distilled model has a significantly larger area than the others. This indicates that the model processed through knowledge distillation has the best overall performance and the highest recognition accuracy for marigolds, which is in line with the expected optimization effect.

As depicted in Figure 7b, a comparative analysis of the mAP@0.5 curves of the four models reveals that during the initial training phase, the YOLOv11n and YOLOv11n-MSEE models exhibit pronounced fluctuations. Conversely, the curves of the pruned model (YOLOv11n-MSEE-Prune) and the distillation model (MPD-YOLO) do not display notable fluctuations, thereby demonstrating superior training stability. It is worth emphasizing that the distillation model commences with a relatively high initial mAP@0.5 value, signifying that it possesses greater stability in the early stages of training. Its performance steadily enhances as the training progresses, characterized by a more robust learning process and exceptional convergence properties. After 100 epochs of training, the performance of all models stabilizes, indicating that the model parameters have converged and the performance has reached its peak.

### 3.2. Comparative Experiments Among Different Models

To evaluate the performance of the YOLOv11n benchmark model for marigold recognition, we compared it against five counterparts: YOLOv5n [27] YOLOv6n, YOLOv8n [28], YOLOv9s [29], and YOLOv10n [30], utilizing an identical dataset. As summarized in Table 1, YOLOv11n achieved a precision (P) of 88.6%, a recall (R) of 86.2%, and a mean average precision at 0.5 IoU (mAP@0.5) of 94.5% for marigold corollas detection. Notably, YOLOv11n attained the highest mAP@0.5 among all evaluated models. While its precision was marginally lower than that of YOLOv9s (89.0%), YOLOv11n demonstrated a substantial advantage in model complexity. Specifically, its model size (5.2 MB) and computational cost (6.3 GFLOPs) were merely 32.2% and 23.6% of YOLOv9s, respectively, representing the lowest among all models. Although YOLOv5n possessed the fewest parameters (2,503,139), YOLOv11n surpassed it in both precision and mAP@0.5 while simultaneously exhibiting a smaller model size and lower computational demands. In summary, YOLOv11n delivers competitive precision and state-of-the-art mAP@0.5 performance for marigold corollas recognition, coupled with minimal model size, parameter count, and computational requirements. This model significantly reduces complexity while maintaining high accuracy compared to alternatives, rendering it suitable for deployment on resource-limited edge devices.

### 3.3. Ablation Experiment

To verify the impact of module improvement, pruning and distillation algorithms on model performance, this study took the marigold recognition task as the benchmark and conducted ablation experiments using the YOLOv11n model to evaluate the effectiveness of the MSEE module, pruning (Prune) and distillation (Distill) algorithms (results are shown in Table 2). After introducing the lightweight MSEE module, precision (P) slightly decreased (by 0.5 percentage points), but the recall rate (R) and mean average precision (mAP@0.5) increased by 0.9 and 0.3 percentage points, respectively, while the number of parameters decreased by 51,816. Further applying the pruning algorithm to the model integrated with the MSEE module, the results showed that the model’s size, number of parameters and computational cost (GFLOPs) were significantly reduced: the model size was reduced to 2.1 MB (only 39.6% of the original model), the number of parameters was drastically reduced from 2,530,531 to 835,336 (equivalent to 33.1% of the original model), and GFLOPs decreased by 3.2 GFLOPs (a 50.8% reduction). Although pruning led to a decrease in accuracy from 88.1% to 86.6%, the recall rate increased by 0.8 percentage points. The model achieved significant compression while maintaining an acceptable accuracy loss, which is beneficial for its deployment on edge devices.

Subsequently, applying the distillation algorithm to the pruned model, the model size, number of parameters and GFLOPs remained unchanged, but the accuracy significantly increased (by 3.2 percentage points), the recall rate slightly decreased, and mAP@0.5 increased from 94.7% to 95.1%,enhancing marigold corollas recognition accuracy Ultimately, after joint optimization with the lightweight module, pruning and distillation algorithms, the model’s precision (P) increased from the baseline of 88.6% to 89.8%, the recall rate (R) slightly decreased by 0.1 percentage points, and mAP@0.5 slightly increased by 0.6 percentage points. In summary, although there was a slight decrease in recall rate, the overall recognition performance of the model improved, and we achieved a significant reduction in model size (size reduced by 59.6%), effectively achieving the lightweight goal of an efficient marigold recognition model.

**Figure 8:**
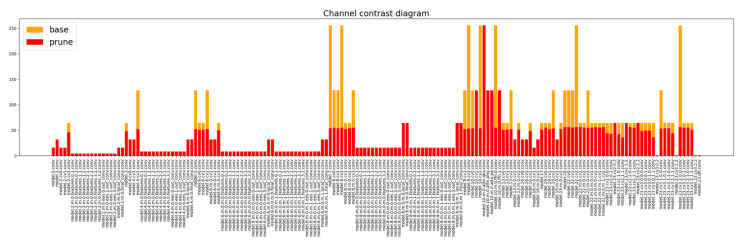
Comparison of model channels.

Figure 8 compares the channel distribution profiles of the model before and after applying the pruning algorithm. The pre-pruning channel density, denoted by the orange region, exhibits significant redundancy and broad spatial coverage, indicative of substantial information redundancy and the presence of numerous low-contribution channels within the original model concerning marigold detection [31]. The structured sparse pruning algorithm effectively eliminated redundant and low-importance channels while selectively preserving those critical for conveying key information. Comparative analysis reveals that the post-pruning feature channels demonstrate markedly enhanced sparsity and selective activation characteristics. This is evidenced by a significant reduction in total channel count and a concomitant concentration and refinement of the feature response regions. Consequently, the pruning process optimally preserves the model’s core representational capacity while substantially reducing its size, parameters, and computational complexity.

**Figure 9:**
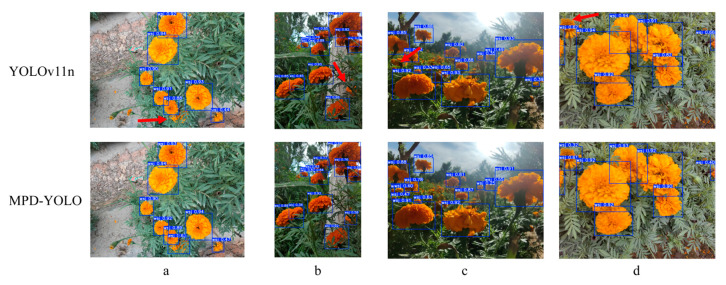
Comparison of test results: (**a**) Front lighting; (**b**) Side lighting; (**c**) Back lighting; (**d**) Adhesion.

As shown in Figure 9, to verify the detection performance of the model in complex marigold scenes, this study conducted comparative experiments under different lighting conditions such as front lighting, back lighting, and side lighting, as well as in large scenes with extensive flower adhesion. The results indicate that both YOLOv11n and its improved model MPD-YOLO can effectively identify marigolds in various environments. However, under back lighting and complex backgrounds, the YOLOv11n model can only recognize flowers with a low degree of occlusion, and in strong light and severely occluded backgrounds, its detection of flowers in dense areas fails. Additionally, YOLOv11n also experiences recognition omissions when dealing with densely adhered and severely occluded flowers (as indicated by the red arrows in Figure 9). In contrast, the improved model proposed in this paper (MPD-YOLO) significantly enhances detection robustness and can effectively identify severely occluded areas and highly densely adhered marigold corollas. This result demonstrates that the improved model has high accuracy in recognizing marigolds under different lighting changes and fully showcases its excellent robustness.

**Figure 10:**
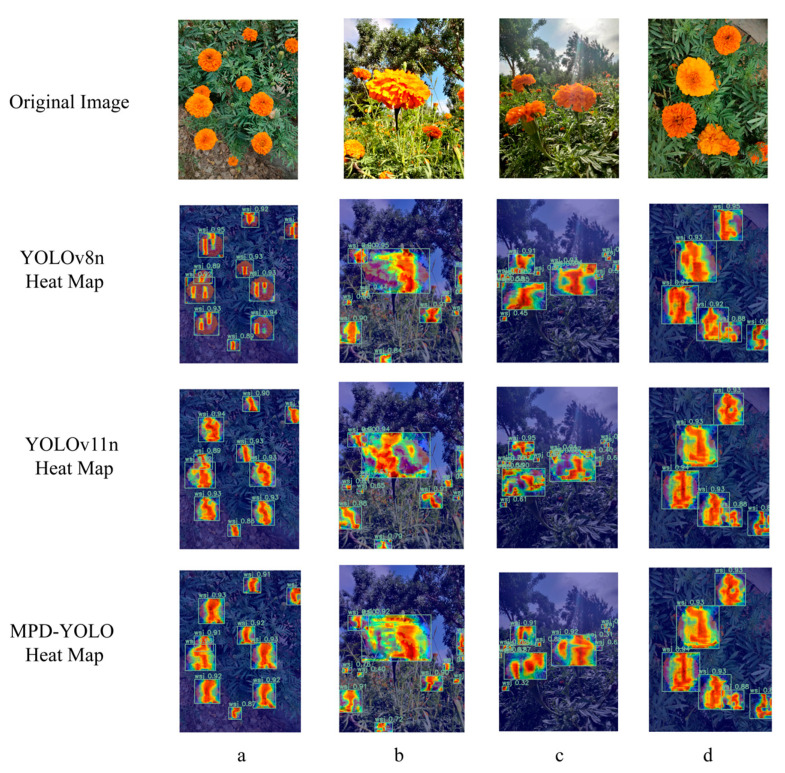
Heatmap of test results: (**a**) Front lighting; (**b**) Side lighting; (**c**) Back lighting; (**d**) Adhesion.

To evaluate the detection performance of the baseline and improved models for marigolds, this study employed Gradient-weighted Class Activation Mapping (Grad-CAM) to generate heat maps. As shown in Figure 10, the heat maps highlight key features through bright regions under three typical lighting conditions (front, back, and side lighting) and in cases where flowers are occluded. The identified marigold corollas are prominently highlighted, creating a sharp contrast with the blue background, which intuitively demonstrates the model’s ability to recognize and locate targets. These visualization results confirm the model’s effectiveness in marigold feature extraction, providing a robust foundation for subsequent continuous picking path planning.

### 3.4. Path Planning Results

Building upon the marigold recognition capabilities of the lightweight MPD-YOLO model, continuous picking path planning was performed. Three classical algorithms—genetic algorithm (GA), particle swarm optimization (PSO), and ant colony optimization (ACO)—were selected for comparative analysis. To mitigate the influence of algorithmic randomness, each algorithm was executed independently for 10 trials; the average path length and average computation time served as evaluation metrics (Table 3). Experimental results demonstrate that both GA and ACO consistently converge to the optimal picking path. However, regarding computational efficiency, GA exhibited significantly higher average time consumption than ACO. Given the high time sensitivity of picking manipulator operation, where prolonged computation directly impacts overall performance, this difference is critical. While PSO eventually reached the optimal solution, its convergence rate was the slowest and solution stability relatively low, indicating poor applicability in this specific context. Consequently, considering the dual criteria of path optimality and time efficiency, ACO was determined to be the optimal benchmark algorithm. Subsequent work will focus on optimizing ACO to further reduce path planning time, thereby enhancing the real-time operational efficiency of the picking manipulator.

**Figure 11:**
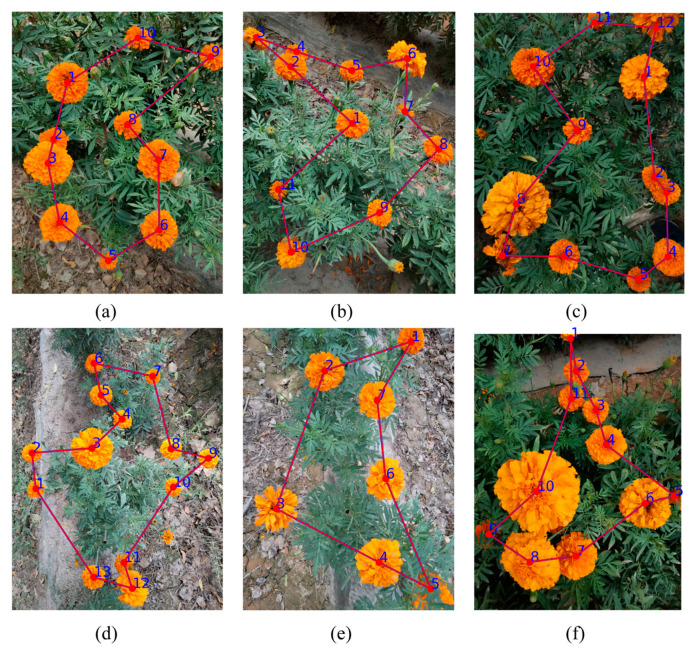
The Optimal Path for Marigold Harvesting: (**a**–**c**) adhere to each other; (**d**) avoid immaturity; (**e**,**f**) occlusion.

Although all three existing algorithms can solve the optimal picking path, their planning time still needs to be optimized. This study enhances the ACO algorithm to significantly reduce path planning time, expediting convergence to optimal solutions and improving picking efficiency. Key innovations include an adaptive parameter tuning mechanism that dynamically adjusts parameters during execution to accelerate computation, and an optimized pheromone update strategy reinforcing pheromone accumulation on superior paths to promote convergence while reducing computational complexity. The improved model has achieved a significant reduction in running time, enhanced robustness, and the dynamic parameter adjustment has reduced the risk of the algorithm getting stuck in local optima (for specific data comparisons, see Table 4). The final planned path is shown in Figure 11 (the planning process path is marked in blue, and the optimal path is displayed in a combination of red and blue to optimize the visual effect). Figure 11a–c indicate that the improved algorithm can effectively identify and plan the picking path of adhered marigolds; Figure 11d shows that the model can avoid picking marigolds that have not fully bloomed; Figure 11e,f verify that the model maintains strong robustness in the presence of occlusions. Most importantly, the average running time of the model has been reduced to just 2.2 s, significantly improving picking efficiency.

## 4. Discussion

This study systematically evaluates the MDP-YOLO model for marigold recognition and the efficacy of its lightweight optimization strategy that incorporates the MSEE module, structured pruning, and knowledge distillation. The proposed approach significantly reduces computational complexity (3.1 GFLOPs) and storage requirements (2.1 MB model size, 835,336 parameters) while effectively maintaining recognition accuracy. The optimized lightweight model achieves 89.8% accuracy under complex lighting conditions (front, back, side). Compared to the lightweight YOLOv7 model proposed by Fan et al. [18] (1,504,000 parameters, lacking reported details on model size and multi-scenario validation), our model demonstrates a substantial parameter reduction, enhancing its suitability for edge deployment. In addition, Fatehi et al. [16] enhanced the recognition accuracy of Damascus rose using a distilled YOLOv9t model; however, their approach relied primarily on distillation and did not incorporate pruning for model lightweighting and size reduction (and model size remains consistently at 4.43 MB). In contrast, the methodology presented in this study applies pruning techniques, maintaining the model size at 2.1 MB and thereby reducing deployment costs. Complex scene evaluations (Figure 10) further confirm the model’s significantly improved robustness against lighting variations, dense petal adhesion, and occlusion, effectively addressing missed detections in densely occluded regions. Critically, the model demonstrates the ability to distinguish and avoid recognizing immature flower buds (unopened marigolds). In addition, the image of marigold can also be processed in combination with large language models to further improve the recognition accuracy [32].

This study addresses the optimization of a lightweight, high-precision recognition model for marigolds and investigates efficient picking path planning based on recognition outputs. Comparative evaluation of traditional path planning algorithms—GA, PSO, and ACO—revealed that ACO exhibited superior computational speed while consistently achieving near-optimal paths, rendering it particularly suitable for real-time harvesting scenarios. To enhance planning efficiency, key modifications to the ACO algorithm were implemented: an adaptive parameter mechanism was introduced to dynamically optimize the search process, and the pheromone update strategy was refined to accelerate convergence towards the optimal path. These optimizations significantly reduced the average path planning time to merely 2.2 s, substantially improving system responsiveness and picking efficiency. Ultimately, the optimized recognition model and the enhanced ACO algorithm were successfully integrated into a comprehensive, practical intelligent marigold picking solution. This integrated system demonstrates significant advantages in recognition accuracy, model efficiency, planning speed, and robustness, providing robust technical support for achieving efficient and precise automated marigold harvesting.

While the lightweight MPD-YOLO model (2.1 MB, 3.1 GFLOPs) has achieved high-precision marigold recognition using high-resolution smartphone imagery, its performance on depth maps from Red Green Blue-Depth (RGB-D) cameras integrated into picking robots remains unvalidated—a current research limitation. Despite this, depth maps offer key advantages: they filter distant vegetation, suppress background interference (potentially boosting detection accuracy), and provide precise 3D spatial positioning for robotic picking arms. A critical issue, however, is that the RGB channel resolution of existing RGB-D cameras is far lower than the high-resolution images used in this study; to address this, the study proposes leveraging an existing high-resolution RGB dataset (2100 training images) to compensate for low-resolution RGB information loss via pre-training or cross-modal fusion, thereby improving RGB-D-based recognition. This scheme further involves: pixel-level alignment of RGB images and depth maps via camera intrinsic calibration (matching MPD-YOLO-detected flower crown bounding boxes to depth maps for 3D coordinates (x, y, z) to avoid 3D planning collisions), adding a depth feature branch in MPD-YOLO’s feature fusion stage (processed via 1 × 1 convolution and MSEE module to output RGB feature attention-weighted results with depth confidence for target prioritization), and adopting cross-modal pre-training of MPD-YOLO on the high-resolution RGB dataset (transferring to low-resolution RGB-D RGB tasks, with depth maps supplementing details). As supported by MPD-YOLO’s robustness in Figure 10, this ensures over 90% detection accuracy even with low-resolution inputs, while the 3D coordinates serve as initial nodes for the improved ant colony algorithm (conducting layered path planning via z-value/height)—fulfilling 3D path planning input priors and upholding the “lightweight–high precision–efficient planning” core logic.

This study addresses the problem of marigold picking path planning in a 2D space. The images for marigold path planning are top views, with the camera almost perpendicular to the marigold plants. Therefore, the optimal picking path planned in the 2D space is also the optimal picking path in the 3D space. However, when the camera is not perpendicular to the marigold plants, the optimal path in the two-dimensional space may be inaccurate. Thus, in the subsequent work, the optimal picking path of marigolds in 3D space will be solved.

## 5. Conclusions

This paper proposes a lightweight marigold recognition model, MDP-YOLO, designed for efficient marigold detection. The model incorporates MSEE into the C3k2 component of YOLOv11n to strengthen feature extraction. Additionally, it is further optimized through pruning and knowledge distillation. Extensive experiments validate its effectiveness, yielding the following key results:

(1) The proposed MDP-YOLO model demonstrates significant advantages in achieving both a lightweight architecture and enhanced recognition performance. Compared with the YOLOv11n baseline, MDP-YOLO achieves a substantial reduction in model size (59.6%) and parameter count (67.7%), with computational complexity of only 3.1 GFLOPs. In terms of recognition performance, the accuracy rate has increased by 1.2%, the mAP has improved by 0.6%, and the recall rate has only slightly decreased by 0.1%. Furthermore, MDP-YOLO achieves a smaller model size than other prominent lightweight YOLO variants, including YOLOv5n (5.3 MB), YOLOv6n (8.7 MB), YOLOv8n (6.3 MB), YOLOv9s (15.2 MB), and YOLOv10n (5.2 MB). This compact size makes it particularly well-suited for deployment on resource-constrained edge devices.
(2) An efficient picking-path planning method was developed. The basic ant colony algorithm was enhanced with an adaptive parameter-adjustment mechanism and an improved pheromone-update strategy, enabling rapid path-planning based on identified marigold positions and providing guidance for subsequent robotic arm harvesting. Under identical conditions, the improved algorithm required only 2.20 s to generate the optimal picking path, substantially shorter than the 18.96 s required by the original algorithm, thereby markedly improving picking efficiency.

To address the challenge of densely clustered marigolds in natural settings, the proposed MDP-YOLO model shrinks network size while retaining high accuracy, enabling efficient deployment on edge devices. Building on the recognition results, an improved ant colony algorithm further reduces the time required for continuous picking-path planning and boosts the efficiency of automated marigold harvesting. These contributions provide theoretical foundations for the development of field-ready marigold-harvesting robots.

## Figures and Tables

**Table 1:** Recognition performance of different models.

Model	P (%)	R (%)	mAP@0.5 (%)	Model Size (MB)	Parameters	GFLOPs
YOLOv5n	87.4	87.1	94.4	5.3	2,503,139	7.1
YOLOv6n	87.8	87.2	94.4	8.7	4,233,843	11.8
YOLOv8n	88.3	85.8	94.3	6.3	3,005,843	8.1
YOLOv9s	89.0	85.2	94.3	15.2	7,167,475	26.7
YOLOv10n	87.2	86.3	94.3	5.2	2,265,363	6.5
YOLOv11n	88.6	86.2	94.5	5.2	2,582,347	6.3

**Table 2:** Ablation experiments.

Baseline Model	MSEE	Prune	Distill	P (%)	R (%)	mAP@0.5 (%)	Model Size (MB)	Parameters	GFLOPs
YOLOv11n	×	×	×	88.6	86.2	94.5	5.2	2,582,347	6.3
YOLOv11n	√	×	×	88.1	87.3	94.8	5.3	2,530,531	6.3
YOLOv11n	√	√	×	86.6	88.1	94.7	2.1	835,336	3.1
YOLOv11n	√	√	√	89.8	86.1	95.1	2.1	835,336	3.1

**Table 3:** Comparison experiment of path planning.

Algorithm	Average Path Length/Pixel	Average Running Time/s	Optimal Path Length/Pixels
PSO	9728.42	35.42	9557.70
GA	9557.70	32.02	9557.70
ACO	9557.70	18.96	9557.70

**Table 4:** Comparison of results of the improved ACO algorithm.

Algorithm	Average Path Length/Pixel	Average Running Time/s	Optimal Path Length/Pixels
Basic ACO	9557.70	18.96	9557.70
Improved ACO	9557.70	2.20	9557.70

## Data Availability

The original contributions presented in this study are included in the article; further inquiries can be directed to the corresponding author.
